# Clinical outcome of patients with inoperable pancreatic cancer treated with FOLFIRINOX or gemcitabine plus Nab‑paclitaxel as a first‑line therapy: A retrospective analysis

**DOI:** 10.3892/mi.2021.8

**Published:** 2021-07-05

**Authors:** Hiroyuki Sakaguchi, Azusa Tanimoto, Shigeki Sato, Naohiro Yanagimura, Chiaki Suzuki, Yohei Takumi, Akihiro Nishiyama, Kaname Yamashita, Shinji Takeuchi, Koshiro Ohtsubo, Seiji Yano

**Affiliations:** 1 Division of Medical Oncology, Cancer Research Institute, Kanazawa University, Kanazawa, Ishikawa 920-0934, Japan; 2 WPI-Nano Life Science Institute (WPI-Nano LSI), Kanazawa University, Kanazawa, Ishikawa 920-0934, Japan

**Keywords:** FOLFIRINOX, gemcitabine, nab-paclitaxel, pancreatic cancer, progression-free survival 2

## Abstract

The present study aimed to evaluate the clinical benefits of leucovorin, 5-fluorouracil, irinotecan and oxaliplatin (FOLFIRINOX) vs. gemcitabine plus Nab-paclitaxel (GnP) as a first-line therapy for patients with inoperable pancreatic cancer. For this purpose, in-house data available for 45 patients who received FOLFIRINOX or GnP as first-line treatment between 2014 and 2019 were retrospectively analyzed. In total, 21 and 24 patients received FOLFIRINOX and GnP, respectively. Although there were no significant differences in the median progression-free survival, the median overall survival was longer in the FOLFIRINOX group than in the GnP group (16.7 vs. 7.2 months). A total of 14 patients received FOLFIRINOX followed by GnP, whereas 3 patients received GnP followed by FOLFIRINOX. All patients who did not switch to second-line therapy owing to poor feasibility were included in the GnP group. The data indicated that patients receiving GnP as first-line therapy were less likely to switch to FOLFIRINOX and, consequently, had a worse prognosis.

## Introduction

The 5-year relative survival rate for all stages combined in pancreatic cancer is lower (9%) than that for other types of cancer reported in the United States (1). Early-stage pancreatic cancer is difficult to detect owing to vague symptoms and its anatomical location, and it often develops into inoperable lesions, such as locally advanced and metastatic cancer (2). Although targeted therapies based on genetic profiles have been developed for inoperable pancreatic cancer, targeted genes including germline BRCA1 or BRCA2 mutations, and oncogenic neurotrophic receptor tyrosine kinase (NTRK)1, NTRK2 and NTRK3 fusions are highly rare (3,4). Thus, cytotoxic chemotherapy remains the mainstream treatment choice for patients with inoperable pancreatic cancer.

Although gemcitabine monotherapy had long been established as a standard, the combination of leucovorin, 5-fluorouracil, irinotecan and oxaliplatin (FOLFIRINOX) and gemcitabine plus Nab-paclitaxel (GnP) has been shown to significantly improve the overall survival (OS), progression-free survival (PFS) and response rate of patients in phase 3 trials (5,6). Compared with gemcitabine, the hazard ratio for mortality in the FOLFIRINOX group was 0.57 [95% confidence interval (CI), 0.45-0.73; P<0.001], while that in the GnP group was 0.72 (95% CI, 0.62-0.83; P<0.001) (5,6). However, each group of patients who participated in these studies had distinct background characteristics; therefore, whether FOLFIRINOX or GnP should be used as first-line chemotherapy remains an open research conundrum. Additionally, there are few reports on the effects of patient background characteristics on treatment options and prognosis in practice.

Liposomal irinotecan (nal-IRI) with 5-fluorouracil and leucovorin following gemcitabine-based therapy has been approved in several countries due to its high antitumor activity and feasibility for use in patients with inoperable pancreatic cancer (7). It can be presumed that combination therapy will be more commonly used following GnP treatment failure, whereas the clinical validity of FOLFIRINOX, which includes irinotecan, 5-fluorouracil and leucovorin, as a second-line therapy following GnP in practice, remains controversial (8,9).

In the present study, the influence of patient characteristics on the selection of either FOLFIRINOX or GnP as a first-line therapy and survival benefits were determined using the data of patients with inoperable pancreatic cancer at Kanazawa University Hospital.

## Patients and methods

### Patients

The present study used the clinical data of patients with inoperable pancreatic cancer treated with modified FOLFIRINOX or Nab-paclitaxel plus gemcitabine as first-line therapy between April, 2014 and January, 2019 at Kanazawa University Hospital. All patients were followed-up once a week or every 2 weeks until they succumbed to the disease. The tumor response was determined in accordance with the Response Evaluation Criteria in Solid Tumors (version 1.1) (10). The present study was approved by the Ethics Board of Kanazawa University (trial no. 2019-178).

### UDP glucuronosyltransferase family 1 member A1 (UGT1A1) gene polymorphism

SN-38 (7-ethyl-10-hydroxycamptothecin), which is an active form of irinotecan, is metabolized by UGT1A1. The gene mutations (UGT1A1*28 and UGT1A1*6) impair its activity, and thus induce severe hematotoxicities in patients treated with irinotecan-based chemotherapy. To analyze UGT1A1 status, genomic DNA was extracted from the peripheral blood leukocytes of the patients.

### Treatment

Modified FOLFIRINOX, consisting of 85 mg oxaliplatin, 400 mg leucovorin, 180 mg irinotecan and 5-fluorouracil administered via continuous intravenous infusion for 46 h at 2,400 mg/m^2^ of body surface area, was repeated every 2 weeks. 5-Fluorouracil through bolus intravenous infusion was excluded in all patients treated with FOLFIRINOX. Nab-paclitaxel plus gemcitabine, consisting of 1,000 mg gemcitabine and 125 mg/m^2^ Nab-paclitaxel of body surface area, was administered on days 1, 8 and 15, and suspended on day 22 every 4 weeks (cycle 1). Chemotherapy dose reduction and delay were performed depending on any observed toxicities such as pneumonitis and neutropenia.

### Statistical analysis

Patient clinical data were analyzed using GraphPad Prism ver. 6.05 (GraphPad Software Inc.). Qualitative variables were compared using Fisher's exact test. OS and PFS were analyzed using the Kaplan-Meier method with a stratified log-rank test. All tests were two-sided, and P<0.05 was considered to indicate a statistically significant difference.

## Results

### Patient characteristics

Between April, 2014 and January, 2019, a total of 45 patients received either FOLFIRINOX (n=21) or GnP (n=24) as first-line chemotherapy at Kanazawa University Hospital. The male to female ratio, age, number of metastatic sites at diagnosis and the type of UGT1A1 gene polymorphism were well-balanced between the 2 groups (Table I). The number of patients with either primary lesions in the head of the pancreas or with an Eastern Cooperative Oncology Group performance status (PS) of 2 was greater in the GnP group than in the FOLFIRINOX group. Additionally, biliary stents were more commonly placed in half of the patients in the GnP group than in the FOLFIRINOX group (Table I).

### Treatment efficacy and adverse events (AEs)

**Figure 1:**
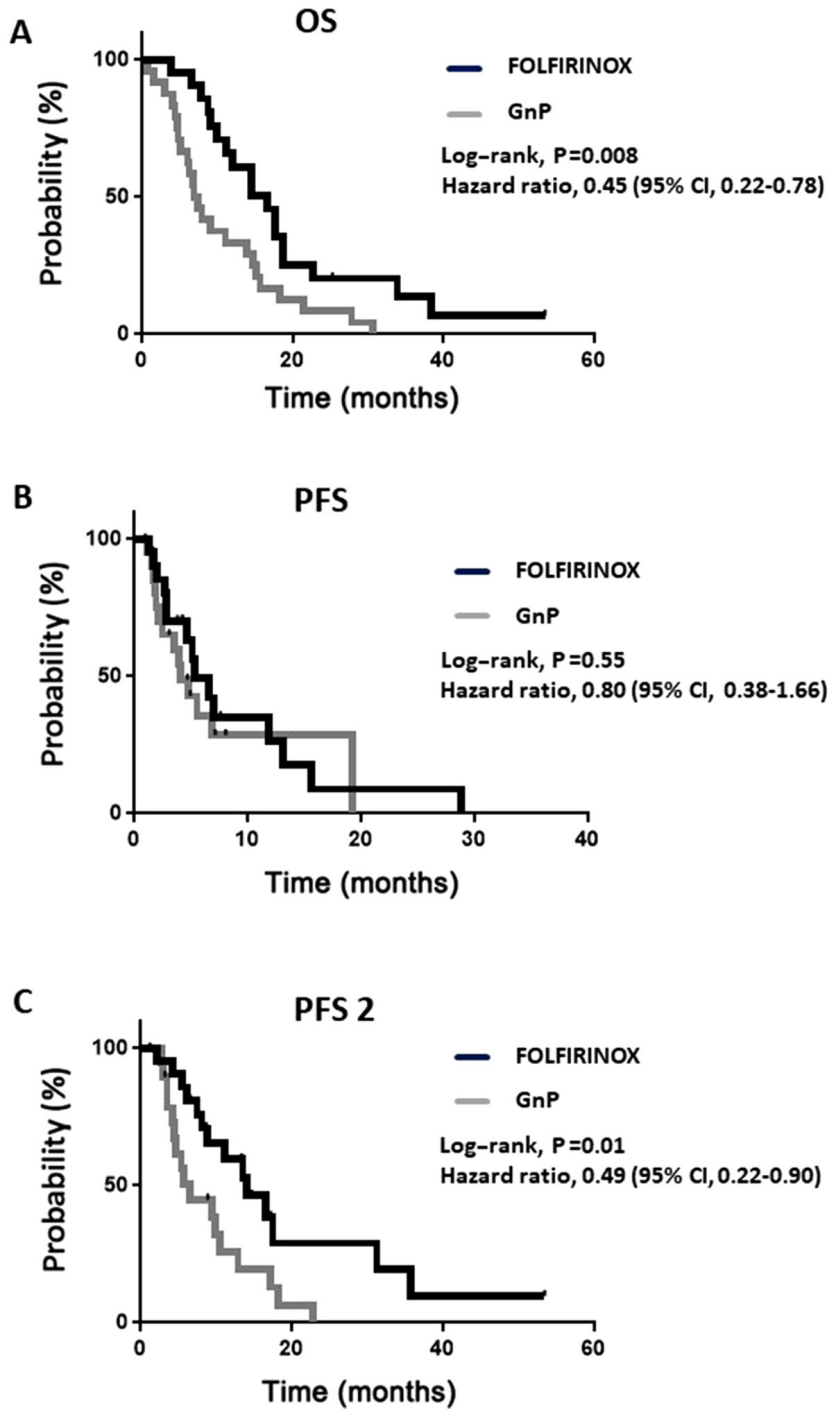
Kaplan-Meier curves of overall survival and progression-free survival in the treatment group. (A) Overall survival; the median was 16.7 months in the FOLFIRINOX group and 7.2 months in the GnP group. (B) Progression-free survival; the median was 5.4 months in the FOLFIRINOX group and 4.0 months in the GnP group. (C) Progression-free survival 2 (the time from the initiation of treatment to second PD or mortality); the median was 14.0 months in the FOLFIRINOX group and 6.5 months in the GnP group. OS, overall survival; PFS, progression-free survival; FOLFIRINOX, combination of leucovorin, 5-fluorouracil, irinotecan and oxaliplatin.

The response rate (partial response) and disease control rate of the FOLFIRINOX group (19 and 85%, respectively) were similar to those of the GnP group (21 and 79%, respectively) (Table II). However, the median OS was longer in the FOLFIRINOX group than in the GnP group (16.7 vs. 7.2 months; hazard ratio for mortality, 0.45; 95% CI, 0.22-0.78; P<0.01) (Fig. 1A). In the PFS analysis, 29 patients received first-line therapy until progressive disease (PD) or mortality, including 67% of the patients in the FOLFIRINOX group and 62% in the GnP group (Table III). In contrast to OS, no significant difference was observed in PFS between the 2 groups (Fig. 1B).

To validate the discrepancy between OS and PFS, PFS 2 was investigated, i.e., the time from the initiation of treatment to second PD or mortality. PFS 2 was significantly longer in the FOLFIRINOX group than in the GnP group (14.0 vs. 6.5 months, 95% CI, 0.22-0.90; P<0.05) (Fig. 1C). The rate of crossover between FOLFIRINOX and GnP was higher in the FOLFIRINOX group (67%) than in the GnP group (12%) (Table III). All patients in the FOLFIRINOX group began second-line therapy, whereas 17% (4 patients) form the GnP group received best supportive care (BSC) instead of second-line therapy (Table III), and three of them had a PS of 2 at baseline. Various AEs caused the cessation of first-line treatment between the 2 groups (Table IV). First-line therapy was discontinued in 16 patients (7 and 9 patients in the FOLFIRINOX and GnP groups, respectively) owing to AEs, and 4 patients had pneumonitis in the latter group.

### Subgroup analyses in the GnP group

**Figure 2:**
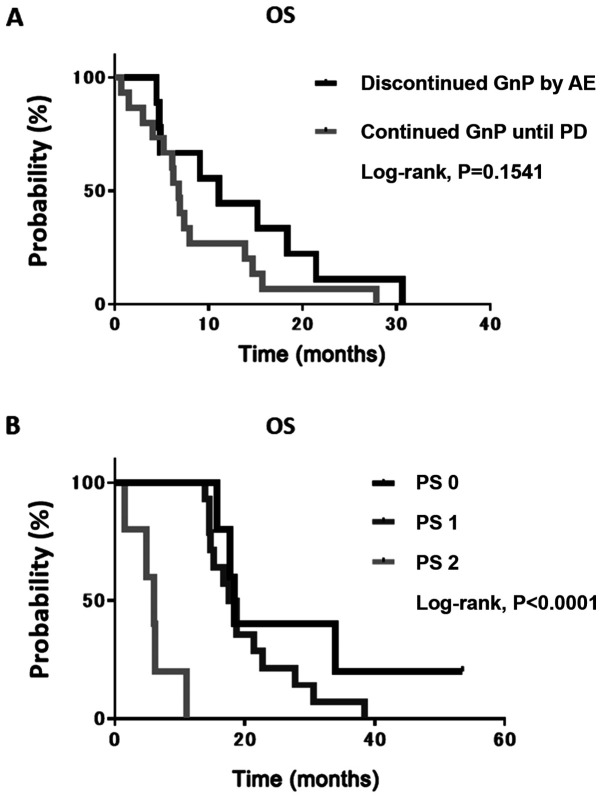
Kaplan-Meier estimates for overall survival in the GnP group, according to cause of therapy interruption and performance status. (A) Overall survival of patients who discontinued treatment due to an AE vs. those who continued treatment until PD. (B) Overall survival of patients with a PS of 0 and 1 vs. 2. AE, adverse event; OS, overall survival; PD, progressive disease; PS, performance status.

Considering that the response rate and PFS did not differ significantly between the 2 groups, it was conceivable that the discontinuation of GnP treatment owing to AEs predicted an unfavorable survival as the AE conflicted with the exclusion criteria of the second-line therapy. To examine this hypothesis, the median OS of patients who continued GnP until PD and in those who discontinued it following AE was determined. However, no significant difference was found between them (Fig. 2A). Thereafter, the present study focused on patients with a lower PS as most switched from first-line therapy to BSC. The median OS of patients with a PS of 2 was significantly shorter than that of patients with either a PS of 0 or 1 (Fig. 2B).

## Discussion

The present study demonstrated that FOLFIRINOX noticeably improved the prognosis of patients with inoperable pancreatic cancer compared with GnP when either of the two therapies was used as a first-line therapy. This result was accounted for by a shorter PFS 2 and more patients with a PS of 2 receiving no second-line therapy, but BSC in the GnP group than in the FOLFIRINOX group. In 41 patients receiving second-line therapy, 14 out of the 21 patients switched from FOLFIRINOX to GnP, whereas only 3 out of 20 patients switched from GnP to FOLIRINOX (Table III). The crossover of the two therapies was far less common in the GnP group than in the FOLFIRINOX group. This is likely to have contributed to the shortening of PFS 2, as some studies have demonstrated that there were no significant differences in median OS between patients with inoperable pancreatic cancer treated with FOLFIRINOX followed by GnP and vice versa (11,12).

In the present study, the low crossover rate in the GnP group was due to AEs, including pneumonitis, cholangitis, and peripheral sensory neuropathy, in the presence of which treatment with irinotecan and oxaliplatin could not be continued. A previous meta-analysis by Pusceddu *et al* (13) suggested that neutropenia and febrile neutropenia were significantly higher in the FOLFIRINOX arm than in the GnP arm as first-line therapy. In the present study, first cycle treatment with G-CSF and the modified regimen without fluorouracil through bolus intravenous infusion curtailed neutropenia, which enabled patients in the FOLFIRINOX group to continue the therapy. A previous retrospective study by Williet *et al* (8) reported that the number of patients treated with GnP following FOLFIRINOX failure was higher than that of patients treated with the reverse sequence, as the former was more feasible than the latter, corresponding to the findings of the present study.

Irinotecan is metabolized and excreted into the gastrointestinal tract through a biliary route following intravenous administration (14). Thus, clinicians tend to avoid the use of irinotecan in patients with biliary obstruction who need biliary stent placement. In fact, the results of the present study demonstrated that more patients with a biliary stent received GnP as first-line therapy than FOLFIRINOX. However, a previous study by Kang *et al* (15) demonstrated that FOLFIRINOX markedly prolonged median stent patency and OS compared with gemcitabine-based chemotherapy, including GnP in patients with pancreatic cancer with stent insertion. FOLFIRINOX as a first-line therapy may improve the prognosis of patients if biliary obstruction and subsequent jaundice are prevented with the biliary stent beforehand.

In recent years, metastatic sites and gene mutations in patients with pancreatic cancer have attracted increasing attention owing to the effectiveness of first-line chemotherapy. Peritoneal carcinomatosis is a poor prognostic factor for patients with pancreatic cancer treated with FOLFIRINOX (16). Moreover, Nab-paclitaxel is expected to have potential in treating peritoneal metastasis due to its pharmacological action, which maintains a high drug concentration in peritoneal lesions (17). Thus, further studies are required to examine the efficacy of GnP in treating pancreatic cancer with peritoneal metastasis. By contrast, Kondo *et al* (18) suggested that homologous recombination repair (HRR)-related gene mutations predicted a favorable prognosis for 17 patients with pancreatic cancer who received oxaliplatin-based chemotherapy. Thus, a prospective trial to investigate the effect of HRR-related gene mutations on the efficacy of FOLFIRINOX is warranted.

In practice, there are differences in the characteristics of patients with inoperable pancreatic cancer that meet the criteria for FOLFIRINOX and GnP. Peixoto *et al* (19) reported that 25 and 45% of patients met the FOLFIRINOX and GnP criteria, respectively, which were in accordance with the pivotal phase III trials (5,6). A common reason for FOLFIRINOX ineligibility was a PS ≥2, corresponding to the finding of the present study that the majority of patients with a PS of 2 received GnP. The prognosis of patients with a PS of 2 resulted in a shorter OS in the GnP group.

In conclusion, the present study reflected real-world data regarding the selection of first-line therapies for patients with inoperable pancreatic cancer. Although patients with a PS of 2 are more likely to be assigned GnP treatment than FOLFIRINOX treatment, the results presented herein revealed that GnP was not established as an effective and feasible treatment for such patients. Additionally, it was found that biliary stent placement impaired the chance of FOLFIRINOX treatment despite the release of obstructive jaundice. These findings may help clinicians select FOLFIRINOX treatment for patients who exhibit good tolerance, while encouraging the development of first-line therapy for pancreatic cancer patients with a worse PS.

## Figures and Tables

**Table I:** Clinical characteristics of the patients in the present study.

	First-line chemotherapy	
Characteristic	FOLFIRINOX, n=21 (%)	GnP, n=24 (%)
Sex		
Male	15(71)	17(71)
Female	6(29)	7(29)
Age, years (median range)	65 (55-75)	67 (53-79)
ECOG PS		
0	7(33)	5(21)
1	13(62)	14(58)
2	1(5)	5(21)
Pancreatic tumor location		
Head	4(19)	13(54)
Uncinate process	1(5)	1(4)
Body	7(33)	5(21)
Tail	7(33)	3(13)
Body and tail	2(10)	2(8)
Biliary stent placement	4(19)	12(50)
Number of metastatic sites		
0	6(29)	5(21)
1	9(42)	11(46)
2	6(29)	6(25)
≥3	0 (0)	2(8)
UGT1A1 gene polymorphism		
Wild	11(53)	8(33)
Heterozygous	9(42)	10(42)
Homozygous	1(5)	5(21)
Unknown	0 (0)	1(4)

FOLFIRINOX, combination of leucovorin, 5-fluorouracil, irinotecan and oxaliplatin; GnP, gemcitabine plus nab-paclitaxel; PS, performance status; UGT1A1, UDP glucuronosyltransferase family 1 member A1.

**Table II:** Chemotherapy response.

Clinical response	FOLFIRINOX, n=21 (%)	GnP, n=24 (%)
Complete response	0 (0)	0 (0)
Partial response	4(19)	5(21)
Stable disease	13(62)	14(58)
Progressive disease	3(14)	5(21)
Not assessable	1(5)	0 (0)
Disease control rate	85%	79%

FOLFIRINOX, combination of leucovorin, 5-fluorouracil, irinotecan and oxaliplatin; GnP, gemcitabine plus nab-paclitaxel.

**Table III:** Selection of therapy following first-line therapy.

	First-line chemotherapy		
Treatment	FOLFIRINOX, n=21 (%)	GnP, n=24 (%)	P-value
First-line treatment interruption for			
Progression	14(67)	15(62)	NSa
Toxicity	7(33)	9(38)	
Second-line chemotherapy			
GEM + Nab-PTX/FOLFIRINOX	14(67)	3(12)	<0.001^a^
Other	7(33)	17(71)	
Best supportive care	0	4(17)	

^a^Fisher's exact test was applied for statistical analysis. NS, not significant. FOLFIRINOX, combination of leucovorin, 5-fluorouracil, irinotecan and oxaliplatin; GnP, gemcitabine plus nab-paclitaxel; GEM, gemcitabine.

**Table IV:** Adverse events leading to the interruption of first-line therapy.

Adverse events	FOLFIRINOX, n=7	GEM + Nab-PTX, n=9
Pneumonitis	1	4
Neutropenia	1	0
Infusion related reaction	1	0
Phlebitis	1	0
Paroxysmal atrial tachycardia	1	0
Gastric hemorrhage	1	0
Diarrhea	1	0
Cholangitis	0	2
Peripheral sensory neuropathy	0	2
Rash maculopapular	0	1

FOLFIRINOX, combination of leucovorin, 5-fluorouracil, irinotecan and oxaliplatin; GEM, gemcitabine

## Data Availability

The datasets used and/or analyzed during the current study are available from the corresponding author on reasonable request.
